# Abscess in the Brain Resulting from Disease of the Ear

**Published:** 1887-05

**Authors:** Thomas Barr

**Affiliations:** Surgeon to the Glasgow Ear Hospital


ABSCESS IN THE BRAIN RESULTING FROM DISEASE OF THE EAR. —*By* THOMAS BARR, M. D., *Surgeon to the Glasgow Ear Hospital.*

It is generally admitted that where abscess in the brain is not due to an injury, it depends usually upon some other pre-existing purulent center, and I shall limit my remarks to that frequent form of abscess in the brain which has its origin in a pre-existing purulent center in the ear.

What is the situation and what is the nature of the ear disease in such cases? The situation is almost invariably, in the first case at all events, in the middle ear. Many of the published records mention the internal ear as the source of the mischief. This is a mistake. No doubt the internal ear as well as the external ear, is sometimes implicated, but then nearly always secondary to the middle ear affection. The air-filled cavities of the middle ear, especially the tympanum, the antrum mastoideum, and the mastoid cells, are primarily and essentially the seats of the mischief in the ear. What is the nature of the disease? It is a chronic purulent inflammation in the lining membrane of these cavities.

The acute form of purulent inflammation of the middle ear is very rarely, if ever, the cause of intracranial disease. This chronic inflammation leads to partial destruction of the tympanic membrane, more or less accumulation of decomposing purulent secretion, and frequently to the formation of granulation-tissue or polypi. The long walls of the middle ear are usually sooner or later involved, either by the occurrence of caries or necrosis, or by hyperostotic or sclerotic changes leading to contraction of the normal cavities of the ear, or to the conversion of the mastoid cells into dense osseous tissue of almost ivory consistence. For months or years, or even a lifetime, the sufferers from this disease go about, usually not at all disconcerted, with purulent septic centers in the interior of their temporal bones. This predisposing cause of cerebral abscess is thus constantly with us in the persons of hundreds and thousands of the community. At the Glasgow Ear Hospital alone, there are usually 300 to 400 persons who annually present themselves suffering from this affection; but it has been noted by various observers, including myself, that, in addition to this predisposing, there is also usually an exciting cause which provokes the mischief in the brain. An injury, such as a blow upon the head or ear, or an irritating influence acting upon the ear, is frequently found to immediately precede the pain in the head or the vomiting which ushers in the graver illness. In nine cases which have come under my own observation, one followed a box on the affected ear, one was due to a blow on the back of the head inflicted with the open hand; in another, the act of diving in the sea, and the consequent rush of cold water into the affected ear, immediately preceded the head-symptoms; in two, the symptoms followed within two or three days of surgical manipulation in the ear, while in the case of the boy who was operated upon by Dr. MacEwen, the exciting cause was probably connected with his fondness for "headers" in playing at football. In a certain proportion of such cases there may have been really a cerebral abscess in a latent form, existing perhaps for months or years, manifesting itself by no definite symptoms until the blow or irritation aroused a new and energetic process in the direction of increased formation of pus or of a fresh inflammation in the walls or in the vicinity of the abscess.

What proportion of cases of abscess in the brain depends upon disease of the ear? There is a general consensus of opinion that ear-disease is distinctly the most frequent cause of cerebral abscess. In the synoptical record of seventy-six cases given by Sir William Gull and Dr. Sutton in their article on "Abscess of the Brain," published in 1872, in Reynolds' *System of Medicine*, twenty-seven cases, or rather more than one-third, were consequent upon ear-disease. In Lebert's article in Virchow's *Archiv.* for 1856, vol. x, based upon eighty cases of cerebral abscess, the proportion given is one in four. But in many of the cases not attributed to the ear, given by these authors, the middle ear had not, it was evident, been examined, while in a goodly number the disease was said to have originated in a blow, which may be quite compatible with its real origin in ear-disease. In the article in Reynolds' *System of Medicine*, there are ten, I think, in which no cause is mentioned. We know that up to the time at which these articles were prepared the hearing organ was very little examined, either in the clinical wards or in the *post-mortem* room. We know also that purulent disease may exist in the middle ear without the knowledge of the friends of the patient, or even of the patient himself. Unless therefore the middle ear had been examined in every one of these cases, we are not in a position to say that these 47 cases in 156 of cerebral abscess give a correct notion of the extent to which ear-disease accounts for cerebral abscess. I am confident that persons die from abscess of the brain, originating in the ear, without the aural origin being known, because the friends of the patient may not be aware of the state of the ear, or may not deem the so-called "running ear" worthy of being mentioned to the medical attendant. Judging from the numerous cases scattered through medical literature and published within the past ten years, during which more close attention has been given to the ear in such cases, and also from the experience of our own hospitals, we are, I think, justified in attributing fully one-half of the cases of abscess in the brain to purulent disease of the ear. As time goes on and as medical men come to use the ear speculum in every case presenting symptoms of cerebral disturbance, the frequency with which ear-disease leads to a fatal issue will be found to be greater than has hitherto been suspected. In looking over the Registrar-General's Annual Reports, I find that the number of deaths in London attributed to otorrhœa, most of which would be due to abscess in the brain, in the year 1882 was eighty-six, and in the eight principal towns of Scotland the number for one year was twenty-six. I think we are entitled to conclude that those numbers, considerable as they are, do not express anything like the real number of victims to this disease who perish annually in London and the eight principal towns in Scotland.

A very important question, especially as bearing upon the operation of trephining the skull in such cases, is as to the most frequent situation of the abscess, and as to the condition of its walls and contents (I speak of course with reference to those originating in the ear). It is unfortunate that in many of the published records these points, especially the state of the walls and contents of the abscess, are not given with that carefulness and minuteness which would have been desirable. In seven cases which came under my own notice, where *post-mortem* examinations were made, the abscess was, in six of them, in the temporo-sphenoidal lobe, above the affected ear. In one of these six there was, in addition, complete occlusion of the lateral sinus, whose walls were converted into a fibrous cord, and there was also a collection of pus in the region of the lateral sinus. In the seventh case the purulent secretion existed between the dura mater and bone, over the posterior surface of the petrous part of the temporal bone. In all my cases the odor from the abscess was extremely fœtid, almost gangrenous, and the pus was generally of a greenish color. In regard to the condition of the walls of the abscess, there was in three of my cases a distinct limiting membrane, encapsuling the abscess and separating it from the neighboring brain-tissue. In the others there was no limiting membrane, the neighboring brain-tissues being soft, broken down, or sloughy. In addition to my own cases I have, in order to gain fuller information especially as to the position of the abscess, gone over the published records of others, in which the abscess in the brain was dependent on ear disease. The whole included 76 cases, of which the abscess was in the cerebrum (generally described as the middle lobe) in 55 cases, or about 73 per cent. of the whole; in the cerebellum, 13; in the cerebrum and cerebellum, 4; in the pons varolii, 2; and in the crus cerebelli in one case. In 9 cases the abscess was said to be encysted, but unfortunately in the greater number there is no information given on this point. As to the condition of the abscess-contents in regard to odor, there is also very little information given in these sixty-nine cases, but when any reference is made to odor, it is described as fœtid. In all the abscess was on the same side as the ear diseased. Rare cases have, it is well known, been recorded, of the abscess being on the opposite side. How is the disease in the middle ear propagated to the interior of the cranium? There are two main directions by which this extension takes place; first, and most frequently, by the roof of the tympanum and antrum mastoideum, to the middle fossa of the cranium, and to the temporo-sphenoidal lobe of the cerebrum above. As shown by the published records of these cases, already mentioned, this is by far the most frequent path by which the disease invades the interior of the cranium. Here the septic purulent focus is separated from the brain above by osseous partition which is never thick, and frequently so thin as to be transparent. It is always perforated with apertures for connective tissue, vessels and nerves. Gaps are not infrequently found, and at these the dura mater and the mucous membrane of the middle ear are in direct contact, while in childhood a very distinct fissure often exists, through which a process of dura mater, with vessels and nerves, passes down and becomes continuous with the mucous membrane lining the tympanum. By simple continuity and contiguity, therefore, without a carious aperture, the diseased process may readily pass upwards to the dura mater and brain, giving rise to a subdural or a cerebral abscess. I show you here four temporal bones where carious apertures will be seen in this situation in all of them.

The second way, in point of frequency and importance, by which the disease in the middle ear extends to the interior of the cranium, is by the inner wall of the antrum and mastoid cells to the dura mater lining the posterior fossa, and to the cerebellum, giving rise to a sub-dural collection of matter, or a cerebellar abscess, or both. Here we have in the deep concavity of the groove for the lateral sinus only a thin osseous partition separating the walls of the sinus from the mastoid cells. It has also the peculiarities mentioned as belonging to the roof of the middle ear—namely perforations, and frequently gaps. In two of the specimens shown by me to-night it will be seen that there are carious erosions and perforations ; but, without such disease of the bone, simple juxtaposition of the lateral sinus with the ear, in suppurative disease of the latter, is a source of great danger. All surgeons know that phlebitis and thrombosis are very apt to form in the vessels of a tissue which is the seat of a purulent inflammation ; and is it surprising that phlebitis and thrombosis should be frequently set up in this large venous trunk, so closely contiguous to, and so connected by blood-vessels with, the diseased part ? Probably some degree of phlebitis of the lateral sinus is not an infrequent complication of purulent disease of the ear. The mischief is, however, in most cases limited to some thickening of its walls, and to a consequent diminution of its lumen.

In either or both of these situations, therefore, disease of the ear most frequently affects the interior of the cranium ; and, while carious apertures are by no means always present there, they are frequently found. They are usually found in the roof of the middle ear when the temporo-sphenoidal lobe is involved, and in the region of the lateral sinus when the cerebellum is the seat of the abscess. In my own cases, five out of seven contained carious apertures; in the other sixty-nine which I have gone over, it is mentioned in twenty-seven cases that caries existed in the petrous or temporal bone, but no precise information is given as to the situation; it was apparent in many of them that this point had not been sufficiently observed. In regard to the mode of extension from the middle ear to the interior of the cranium, there is no doubt that in some, although not in many, the purulent contents of the middle ear, pent up and hindered by some obstruction from escaping through the outer channels of the ear, burst through a carious aperture or natural foramen, or a gap, into the middle or posterior fossa, burrowing between the dura mater and bone, or, if the dura mater has been previously softened and perforated, making its way into the arachnoid space, or into the tissue of the brain itself. But probably more frequently, even where caries does exist, there is simply a direct transmission, by vessels and lymphatics, of the septic inflammation from the cavities of the middle ear to the dura mater and its sinuses in the first place; and then, by contact, an inflammation of a similar character affects the pia mater and brain. In those cases—which, I think, are not very frequent—where the parts between the brain-abscess and the middle ear seem to be healthy, the propagation probably takes place by the immigration of bacteria from the purulent focus in the ear along the lymphatics surrounding these branches of the internal auditory artery which anastomose with the vessels of the pia mater, and thus reach the interior of the brain. Multitudes of bacteria have been found in a cerebral abscess a few hours after death. Dr. James Alexander

Adams suggests that the bacteria reach the brain through the veins ; that, where the lateral sinus is occluded, there is a backward current of venous blood. In this way, it is suggested, bacteria attached to thrombi carried from the ear by the veins which pass into the sinuses of the dura mater find their way into the interior of the brain.

There is no doubt that pus from an abscess in the brain sometimes makes its way into the ear, probably through the roof of the middle ear. In the case in which Dr. MacEwen operated there was unquestionably a partial escape of the matter from the brain into the ear for two or three days before the operation, but this was attended by no improvement in the symptoms. Some of the older writers describe an "otorrhœa cerebralis," in which the abscess in the brain was thought to be the primary condition, the discharge from the ear being secondary, and coming directly from the abscess in the brain. The opinion now generally held is that in all such cases the ear-disease is the primary condition and the cause of the brain-abscess. I was very much interested, however, in being informed by Dr. McLeod that he saw a case of cerebral abscess in the frontal lobe due to an injury, and associated with necrosed bone over the seat of the abscess, in which the matter was proved to have made its way along the base of the skull to the roof of the external auditory canal, through which it penetrated, and found exit by the outer orifice of the ear.

Finally, with respect to treatment, the recent cases of Mr. Barker, Professor Greenfield, and Dr. MacEwen have inaugurated a new era in the previously hopeless, dismally hopeless, cases of cerebral abscess. Previous to the cases of these gentlemen, I know of only two recorded instances of recovery after opening the skull for cerebral abscess, both due to ear-disease. One was reported by C. Tauckenbrod, of Hamburg, in the *Archives of Otology*, vol. xv, Nos. 2 and 3, 1886. In this case, the mastoid process was opened first, and a quantity of fœtid caseous matter washed out. The opening in the wall of the skull was made by chiseling backwards and upwards for a distance of about three centimeters from the opening in the mastoid. A small rough place was found in the bone, and here a piece was chiseled off, and the dura mater exposed. There was pus between the dura mater and bone ; and, on puncturing the brain at this part, a cupful of matter escaped. The abscess-cavity was syringed with a solution of corrosive sublimate. Ultimate recovery took place, and the patient followed his occupation of architect, his only drawback being a certain degree of word-forgetfulness, aphasia having been a striking feature in the symptoms previous to the operation.

The other case was by Dr. Schondorff, described in the *Monatschrift für Ohrenheilkunde*, 1885, No. 2. Here there was a fistular orifice in the skull leading to the brain; where the abscess existed. It will be seen that these two cases are different from the recent ones in this important respect —that, in the former, there were affections of the bones of the skull indicating the probable seat of the matter, while in the latter no such indications existed. It is quite clear that, in the future, persons suffering from abscess in the brain, dependent upon ear-disease, should not be left to die as they have been in the past without an effort being made by opening the interior of the cranium to reach and drain the abscess ; and an important question—which, perhaps, cannot be settled until further experience has been gained—is, how can the abscess be best reached, and how can it be most efficiently drained?

Speaking of abscess in the temporo-sphenoidal lobe, the most common form, it might be said that if we could penetrate the floor of the middle fossa of the skull through the roof of the middle ear, we would get access to the most dependent part of the abscess in the brain, or to any collection of matter between the bone and the dura mater; such a method would also imitate that by which Nature sometimes attempts to get rid of the matter. This plan has been attempted and it failed.

A case is reported by Dr. Sutphen, of America (*Archives of Otology,1884*), in which an opening was drilled with a trocar in the roof of the tympanum, and no pus escaped, although after death a large collection was found in the brain above. The great objection to this mode of operating is the difficulty of making and maintaining a sufficiently large opening for the escape of the pus, in such a narrow space as the tympanic cavity, made still more narrow by inflammatory swelling. The plan pursued by Dr. MacEwen seems to be the most practicable, namely, to open the skull about two inches above the ear, and, when the abscess is found, to make another opening lower down, nearly on a level with the floor of the middle fossa of the skull. If a current of fluid can be made to pass from the upper to the lower openings, thorough cleansing would be ensured. I am not sure of the desirability of invariably opening into the mastoid cells as preliminary to the larger operation. If a case presented indisputable signs of intracranial mischief, and if there was neither redness, swelling, nor tenderness over the mastoid process, I question the propriety of opening into the latter. No doubt we would often find caseous matter, in examining the cavities of the middle ear after death, in any person who has suffered from chronic suppurative inflammation ; although his death may have had no relation thereto, caseous matter would be found. In a person who has possibly meningitis or a cerebral abscess, it is questionable practice to submit the cranium, without a very important reason, to the concussion caused by striking a chisel with a mallet, this having been hitherto regarded as the safest mode of opening the mastoid cells. Again, if the lateral sinus is far forward, which it not infrequently is, great care indeed is required to avoid doing serious injury.

In a temporal bone from a case of cerebral abscess which occurred in the Ear Hospital in September last, it was seen that only one-twelfth of an inch intervened between that part of the outer surface of the bone where it is recommended that the perforation should be made and the lateral sinus. If an attempt had been made to trephine or drill the bone at this position, there would have been great danger of fatally wounding the lateral sinus.

I may conclude with one remark as to preventive treatment. It is this—that when every member of our profession is sufficiently impressed with the importance of chronic suppurative inflammation of the middle ear, and prepared to efficiently treat this disease in all its stages, the occasion for this operation will probably seldom arise.—*British Medical Journal.*

